# Genome-wide localization of histone variants in *Toxoplasma gondii* implicates variant exchange in stage-specific gene expression

**DOI:** 10.1186/s12864-022-08338-6

**Published:** 2022-02-14

**Authors:** Sheila C. Nardelli, Natalie C. Silmon de Monerri, Laura Vanagas, Xiaonan Wang, Zoi Tampaki, William J. Sullivan, Sergio O. Angel, Kami Kim

**Affiliations:** 1 Department of Pathology, Albert Einstein College of Medicine, Bronx, NY 10461 USA; 2 Present address: Instituto Carlos Chagas/Fiocruz-PR, Curitiba, PR CEP 81.350-010 Brazil; 3 Department of Medicine, Albert Einstein College of Medicine, Bronx, NY 10461 USA; 4 Present address: Pfizer Inc, Pearl River, NY 10965 USA; 5 Laboratorio de Parasitología Molecular, Instituto Tecnológico Chascomús (INTECH), Consejo Nacional de Investigaciones Científicas (CONICET)-Universidad Nacional General San Martin (UNSAM), Chascomús, Argentina; 6 Department of Pathology, University of Cambridge, Cambridge, UK; 7 Present address: School of Public Health, Shanghai JiaoTong University, School of Medicine, Shanghai, China; 8 Department of Pharmacology and Toxicology, Indiana University School of Medicine, Indianapolis, Indiana, 46202 USA; 9 Department of Microbiology & Immunology, Albert Einstein College of Medicine, Bronx, NY 10461 USA; 10 Department of Internal Medicine, Division of Infectious Disease and International Medicine, Morsani College of Medicine, University of South Florida, Tampa, FL 33612 USA

**Keywords:** Histones, Parasites, *Toxoplasma*, ChIP-seq, Apicomplexa, Epigenetic, Transcriptional regulation

## Abstract

**Background:**

*Toxoplasma gondii* is a protozoan parasite that differentiates from acute tachyzoite stages to latent bradyzoite forms in response to environmental cues that modify the epigenome. We studied the distribution of the histone variants CenH3, H3.3, H2A.X, H2A.Z and H2B.Z, by genome-wide chromatin immunoprecipitation to understand the role of variant histones in developmental transitions of *T. gondii* parasites.

**Results:**

H3.3 and H2A.X were detected in telomere and telomere associated sequences, whereas H3.3, H2A.X and CenH3 were enriched in centromeres. Histones H2A.Z and H2B.Z colocalize with the transcriptional activation mark H3K4me3 in promoter regions surrounding the nucleosome-free region upstream of the transcription start site. The H2B.Z/H2A.Z histone pair also localizes to the gene bodies of genes that are silent but poised for activation, including bradyzoite stage-specific genes. The majority of H2A.X and H2A.Z/H2B.Z loci do not overlap, consistent with variant histones demarcating specific functional regions of chromatin. The extent of enrichment of H2A.Z/H2B.Z (and H3.3 and H2A.X) within the entire gene (5’UTR and gene body) reflects the timing of gene expression during the cell cycle, suggesting that dynamic turnover of H2B.Z/H2A.Z occurs during the tachyzoite cell cycle. Thus, the distribution of the variant histone H2A.Z/H2B.Z dimer defines active and developmentally silenced regions of the *T. gondii* epigenome including genes that are poised for expression.

**Conclusions:**

Histone variants mark functional regions of parasite genomes with the dynamic placement of the H2A.Z/H2B.Z dimer implicated as an evolutionarily conserved regulator of parasite and eukaryotic differentiation.

**Supplementary Information:**

The online version contains supplementary material available at 10.1186/s12864-022-08338-6.

## Background

Eukaryotic cells have specialized macromolecular complexes that organize and pack DNA inside the nucleus. These multiprotein complexes include histones (H2A, H2B, H3, H4) and non-histone proteins. DNA is wrapped around an octamer formed by two copies of each histone to form the nucleosome. Nucleosome assembly is initiated with the association of tetrameric H3-H4 with DNA, followed by two dimers of H2A-H2B. A fifth histone, H1 or linker histone, helps to pack 20 bp of additional internucleosomal DNA [1].

Histones are small, basic proteins that represent about 50% of the total volume of chromatin [2, 3]. The interaction of histones with DNA and non-histone proteins affects all nuclear processes, including replication, transcription and DNA repair. Eukaryotic cells have evolved three main strategies to regulate nucleosomes. First, ATP-dependent chromatin remodeling complexes use the energy from ATP hydrolysis to remodel chromatin by replacing or sliding nucleosomes, thus affecting their interaction. Second, histones are extensively modified with posttranslational modifications (PTMs). These PTMs regulate DNA-related processes in a positive or negative way, influencing the interaction between the histones and DNA or recruiting protein factors involved in gene expression or gene silencing. Finally, cells replace canonical histones with histone variants [4, 5] . Unlike canonical histones, whose synthesis is coupled to DNA replication during S-phase, histone variants are typically constitutively expressed through the cell cycle [6] . The physical properties of histone variants can change chromatin structure, or histone variants may localize to specific regions of the genome to confer specialized properties to chromatin.

*Toxoplasma gondii* chromatin structure has been implicated as an important regulator of gene expression. *T. gondii* is an opportunistic parasite that infects between 10 and 90% of the population depending on the country [7]. Although *T. gondii* infection is asymptomatic in healthy people, untreated clinical toxoplasmosis is lethal in immunocompromised individuals such as transplant or HIV patients. In addition, toxoplasmosis is an important cause of congenital defects or fetal demise when women become infected during pregnancy. In humans and other intermediate hosts, *T. gondii* tachyzoite stages are associated with acute disease, while bradyzoites within tissue cysts persist in a chronic phase. The ability of the parasite to persist as a latent bradyzoite form is critical for disease pathogenesis and the developmental transition from tachyzoite to bradyzoite is likely driven by epigenetic processes involving histone PTM and histone localization [8].

*T. gondii* expresses four canonical histones, represented by single copy genes, except for H2B that is encoded by two separate genes on different chromosomes [9, 10]. H2Ba is expressed mainly in tachyzoite forms while H2Bb expression is restricted to the sexual stages [11, 12] . H4 does not have a variant, but interacts with H3 and its variants H3.3 and centromeric CenH3 [13]. H2A has two variants: H2A.X, which shares 91% identity with canonical H2A, and H2A.Z, which is considered the most conserved histone variant across species [14] . To date, no histone H1 has been identified in Apicomplexa.

H3.3 differs from H3 by just four amino acids in *T. gondii* (RY instead of KF at position 54–55; QAIL in H3.3 and SAVL in H3 in positions 88–91) [15]. Although there are only minor sequence differences between H3.3 and H3, these few differences can result in different roles in gene activation [16]. CenH3 is a conserved variant necessary for kinetochore formation in several organisms, and TgCenH3 is located at centromeric regions of *T. gondii* [13]. *T. gondii* centromeres are arranged in a single apical cluster, represented by a dot that is attached to the centrocone during all cell cycle by immunolocalization [13].

*T. gondii* H2A.X has the conserved SQ(E/D) φ motif important for DNA repair (φ represents a hydrophobic amino acid and S is a serine that is phosphorylated in response to DNA damage). Serine 132 of H2A.X is phosphorylated in tachyzoite forms of *T. gondii* and plays a role in DNA repair [14, 17]. TgH2A.Z shares 82% identity with human H2A.Z, though TgH2A.Z has an additional 20 amino acids in the N-terminal region. By Chromatin Immunoprecipitation followed by qPCR (ChIP-qPCR), TgH2A.Z was detected at the promoter regions of active genes, a conserved characteristic of H2A.Z in most organisms, whereas TgH2A.X was associated with silent genes [14].

H2B.Z (previously known as H2BV), is a variant unique to Apicomplexa [12, 18, 19] that has been proposed to be the major histone H2B in *T. gondii* tachyzoites. H2B.Z interacts with H2A.Z but not H2A.X [14]. Mass spectrometry analysis of *T. gondii* tachyzoites revealed that both H2B.Z and H2A.Z are hyperacetylated in their N-terminal domains, a classic hallmark of active chromatin [20], and both are also substochiometrically ubiquitinated [21]. By contrast, few or no acetylation marks have been detected on canonical H2A, H2B or H2A.X [20].

In this study, we surveyed the genome-wide location of histone variants across the *T. gondii* genome, using Chromatin Immunoprecipitation. Our findings implicate histone variant exchange in regulation of stage-specific transcription and expand our understanding of histone variants in eukaryotes.

## Results

### Localization of histone variants is not uniform within nuclei

**Fig. 1:**
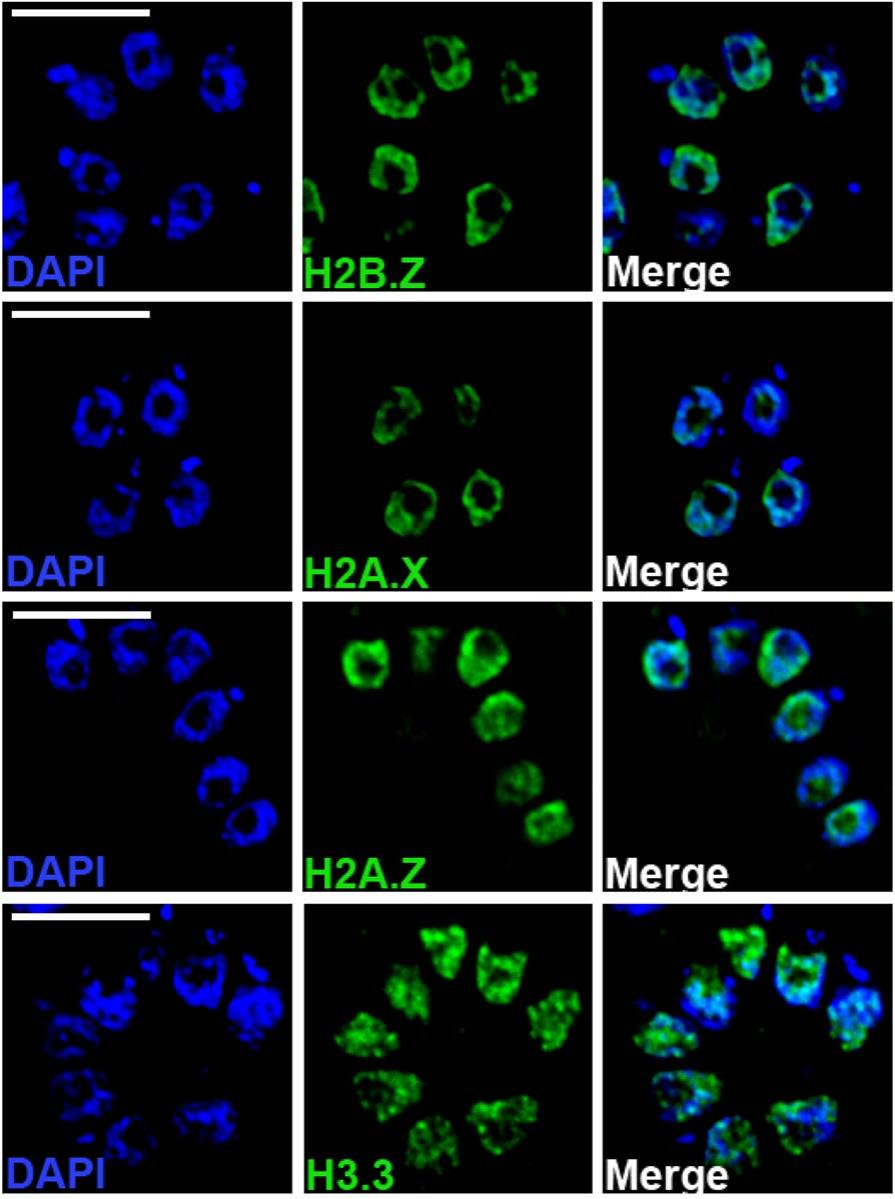
Non-uniform localization of histone variants in *T. gondii*. Distribution of histone variants in the nucleus of tachyzoite forms. HFF cells were grown on glass slides and infected with tachyzoites for 24 h. Slides were stained with DAPI and antibodies against anti-H2AZ, anti-myc (for H2AX-myc and H2BZ-myc) or anti-HA (H3.3). Images were collected along a Z-series and processed for three-dimensional deconvolution using similar signal thresholds for each of the antibodies. The small extranuclear DAPI staining in some cells labels the genome of the apicoplast. The merged figures correspond to the overlay of DAPI (blue) and histone variants (green). The scale bars correspond to 5 μm

We confirmed the location of histones variants in the nuclear compartment by labeling intracellular RH strain tachyzoites with monospecific polyclonal antibodies recognizing *T. gondii* histone variants or endogenous epitope-tagged proteins, and imaging by deconvolution fluorescence microscopy (Fig. 1). Comparison of localization patterns with the staining intensity of DAPI allowed us to examine the presence of the histone variants in dense and less dense chromatin. Regions of “heterochromatin” stain more intensely with DAPI than the “euchromatic” domains. None of the histone variants localized exclusively to heterochromatic or euchromatic chromatin. H3.3 showed punctuate distribution, generally opposite to DAPI staining, while H2B.Z and H2A.Z exhibited a limited degree of co-localization with dense chromatin (Fig. 1). H2A.X localized most intensely with regions of compact chromatin, but not exclusively to heterochromatin. None of the histones colocalized with DAPI within the apicoplast, a remnant organelle of endosymbiosis present in many Apicomplexan parasites.

### Native ChIP-chip and ChIP-seq in *T. gondii*

ChIP followed by tiling microarray hybridization (ChIP-chip) or high throughput sequencing (ChIP-seq) are widely used for studying genome-wide protein-DNA interactions. In previous work, we applied ChIP-chip to *T. gondii* and showed that the distributions of epigenomic modifications on chromatin are conserved in protozoa [22]. We standardized native chromatin immunoprecipitation (N-ChIP) for *T. gondii* using native DNA without crosslinking digested with Micrococcal nuclease (MNase) followed by next generation sequencing [23]. The digested fragments of around 150–200 bp represent nucleosomal DNA, as verified by nucleosome mapping of input DNA. As controls, we performed N-ChIP with antibodies specific for H3K4me3 and CenH3. Using N-ChIP-seq, we observed similar locations to those in our previous studies using cross-linked DNA [13, 22].

**Fig. 2:**
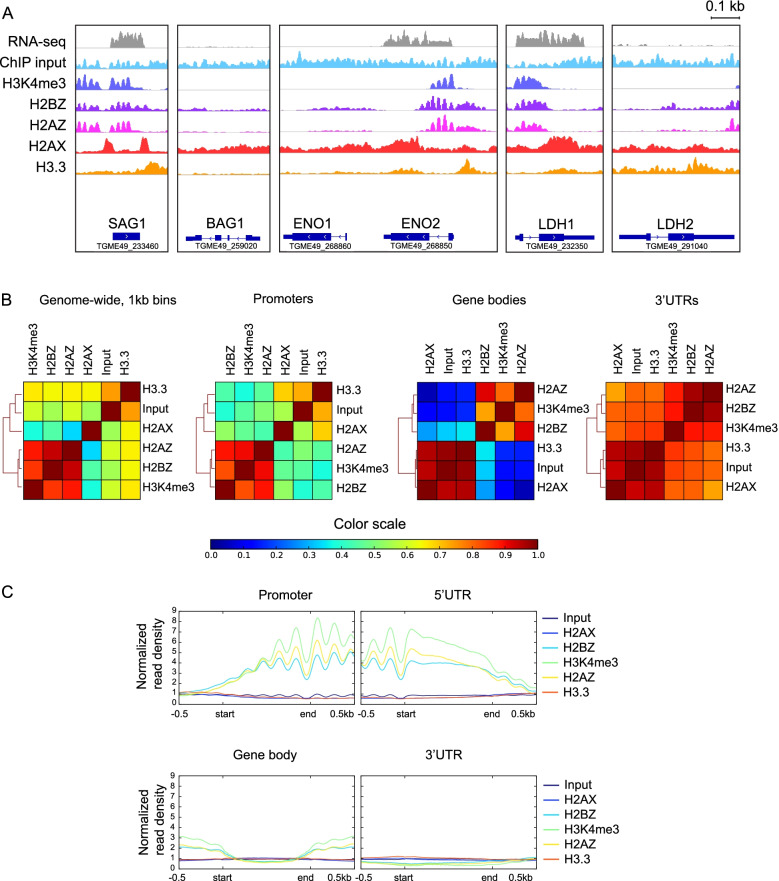
Histone variants have distinct localization genome-wide. **A**. Pileups of histone variant ChIP-seq reads mapping to genes of interest. Note, individual nucleosomes can be resolved from this data. Genes shown are tachyzoite genes *SAG1*, *ENO2* and *LDH1* and bradyzoite genes *BAG1*, *ENO1* and *LDH2*. **B**. The overall similarity in ChIP-seq profiles was compared by calculating Pearson correlation coefficients genome-wide or for different genomic features including promoters, gene bodies and 3′ UTRs (as defined by www.toxodb.org annotations). Correlations were visualized in a clustered heatmap (generated using deeptools http://deeptools.readthedocs.io); color indicates correlation coefficient as shown in color key. Peaks for each variant represent the intersection of peaks from each biological replicate. **C**. Average ChIP-seq profiles of each histone variant on different genomic features. Start and end denote boundaries of each feature, with 500 bp upstream and downstream flanking sequence either side. ChIP-seq read pileups were normalized by scaling to library size

To study the genome-wide localization of histone variants in *T. gondii*, both ChIP-seq and ChiP-chip were performed using antibodies described above and epitope-tagged lines in the Type I RH strain background. A summary of experiments and platforms used is shown in additional file 1, along with a summary of how the data was processed and analyzed. Histone variants display distinct localization patterns that differ over genes with varying expression profiles such as LDH1 (tachyzoite) and LDH2 (bradyzoite), ENO1 (bradyzoite) and ENO2 (tachyzoite) or SAG1 (tachyzoite) and BAG1 (bradyzoite) (Fig. 2A). ChIP-seq showed enrichment over the same genes as ChIP-chip (Fig. 2A and additional file 2A) and positive correlation between H2A.Z, H3K4me3 and H2B.Z ChIP-chip and ChIP-seq replicates (Pearson’s r > 0.75) (additional file 2). Correlations between ChIP-seq replicates are shown in additional file 3.

### Localization of histones and histone-variants across the genome

The correlation between histone variant locations was examined by correlating the read densities of each histone variant ChIP-seq dataset (Fig. 2B). H2A.Z and H2B.Z signals are strongly correlated, consistent with their reported association as a dimer in nucleosomes [14]. H3K4me3 also correlated with H2A.Z and H2B.Z. Histones H2A.X and H3.3 distributions are positively correlated, and weakly negatively correlated with H2A.Z/H2B.Z (Fig. 2B). This supports the hypothesis that in *T. gondii*, H2A.X and H2A.Z are found in different nucleosomes [12, 14]. The same pattern was observed at promoters, 5’UTRs, and gene bodies (Fig. 2B). Normalized read densities at promoters, 5’UTRs, gene bodies and 3’UTRs are shown in Fig. 2C. At 3’UTRs, little signal was detected for any histone variant (Fig. 2C).

In tachyzoites, a majority of genes were marked by H3K4me3 (~ 75%), H2A.Z (~ 80%) and H2B.Z (~ 90%) (additional file 4A). H3K4me3, H2A.Z and H2B.Z peaks localize predominantly to promoter regions; H3.3 and H2A.X mark ~ 75% and ~ 45% of genes respectively and localize to regions upstream or downstream or overlapping of mapped TSS (additional file 4B, C, D). Together, these findings imply that nucleosomes at promoter regions and gene bodies have distinct compositions. Promoter regions are marked by nucleosomes containing H2A.Z, H2B.Z and presumably canonical H3, while gene bodies are occupied by nucleosomes containing H2A.X and H3.3 and possibly canonical H2Ba [11].

Since H3K4me3 colocalizes with H2A.Z/H2B.Z, these data imply exchange of H2Ba/H2A or H2Ba/H2A.X dimers, with double variant dimer composed of H2A.Z/H2B.Z [14, 24, 25] at promoter regions of active genes.

### Centromeres and telomeres have unique histone compositions

**Fig. 3:**
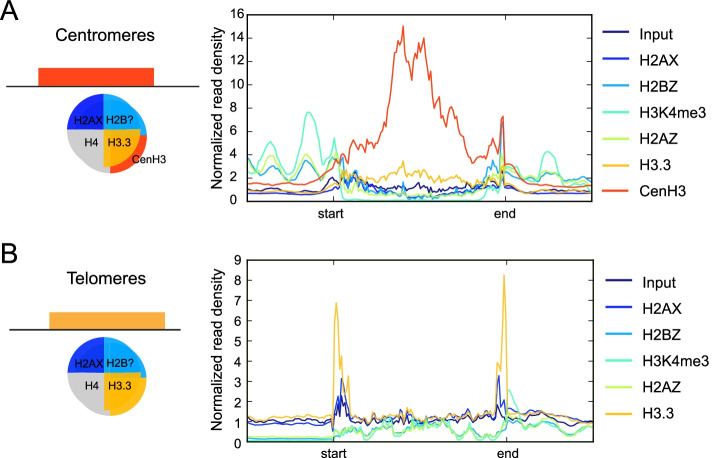
Composite profiles of histone variant distribution at centromeres and telomeres. Profiles were generated by averaging the ChIP-seq read density over annotated genomic loci for the histone variants as indicated by the colored lines. ChIP-seq profiles were normalized by scaling to 1x library size. Start and end denote boundaries of each feature, with 500 bp upstream and downstream flanking sequence either side. **A**. Centromeric regions. **B**. Telomeric regions

Centromeres and telomeres have distinct functions and are often associated with altered histone composition. We examined histone variant localization at these specialized regions. CenH3 localizes almost exclusively to centromeric regions and is accompanied by H3.3, while H2A.X, H2A.Z and H2B.Z are absent from centromeres (Fig. 3A). CenH3 localized to 13 different loci and was absent from Chromosome VIIb, consistent with the revised genome of *T. gondii* containing 13 chromosomes [26]. Further work is required to determine whether CenH3 and H3.3 are part of the same nucleosome within a double variant heterodimer of CenH3 and H3.3, or if *T. gondii* centromeres consist of interspersed CenH3 and H3.3-containing nucleosomes that may in turn be enriched at different phases of the cell cycle [27].

At telomeres and telomeric associated sequence (TAS), CenH3 is absent and H2A.X and H3.3 are enriched (Fig. 3B), suggesting that they may play a role in telomeric and TAS maintenance [11]. As in other species, *T. gondii* telomeres and TAS are notable for repetitive DNA sequences [28] making accurate genome annotation and assembly difficult. Nevertheless, H3.3 and H2A.X showed high levels of signal at the ends of at least 10 of the 14 chromosomes (data not shown), suggesting a role in protecting the extremities of telomeres. *T. gondii* telomeres are also associated with the H3K9me3 repressive mark in ChIP-Seq [29] and the HP1 orthologue Chromo 1 [30]. H2A.X, along with H3.3, was also found in lower levels spread through the chromosomes, usually opposite to H2A.Z (Fig. 2A).

### H2A.Z and H2B.Z mark promoters of active genes

**Fig. 4:**
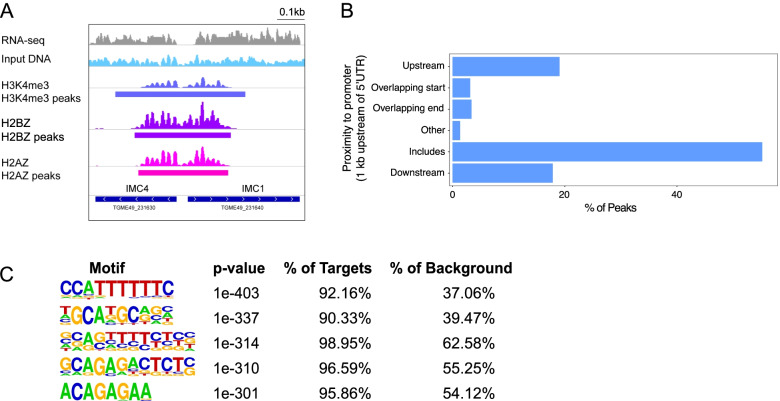
H3K4me3, H2A.Z and H2B.Z overlap at promoters. **A.** Genome browser view (visualized using IGV) of H3K4me3, H2A.Z and H2B.Z ChIP-seq read density profiles at two genes (*IMC1* and *IMC4*) with opposing orientations (bottom track). RNA-seq from *T. gondii* RH strain tachyzoites is shown in grey (from [31]). Peaks called using the peak-calling algorithm MACS2 are shown under read density profiles. **B**. Location of combined H3K4me3, H2A.Z and H2B.Z peaks in relation to promoter regions (defined as 1000 bp upstream of 5’UTR start, defined from RNA-seq data on tachyzoites [31]). **C**. Sequence motifs identified by HOMER that are significantly enriched in genomic regions where H3K4me3, H2A.Z and H2B.Z overlap

H2A.Z has a conserved function in transcriptional activation [28]. In *T. gondii*, 98% of H3K4me3 peaks colocalize with H2A.Z and H2B.Z in or around promoter regions (Figs. 2 and 4 A, B). Even in the asynchronous tachyzoite cultures we sampled, strong nucleosome positioning was evident at the 5′ region of active genes. Several nucleotide motifs were enriched in these regions (Fig. 4 C), suggesting that H2A.Z and H2B.Z are positioned with H3K4me3 and H4ac in promoter regions [22] that recruit macromolecular complexes consisting of transcription factors and chromatin modifying enzymes.

Distinct profiles of histone variant coverage were observed on mRNA compared to rRNA or tRNA (additional file 5A). rRNA genes, which are highly transcribed in tachyzoites, were relatively depleted of histones, whereas tRNA genes were flanked by H3K4me3. The presence of H3K4me3, H2A.Z and H2B.Z at promoters and 5’UTR was associated with gene expression (additional file 5B), although there did not seem to be significant differences in ChIP-seq signal when genes with low, medium or high levels of expression (determined from RNA-seq data [31]) were compared. H3K4me3, H2A.Z and H2B.Z were not enriched in the 5′ regions of genes whose expression was in the lowest expression quintile. Normalized read densities were similar for all variants at G1-regulated or SM-regulated genes (additional file 5C).

### H2A.Z and H2B.Z mark gene bodies of silent genes

**Fig. 5:**
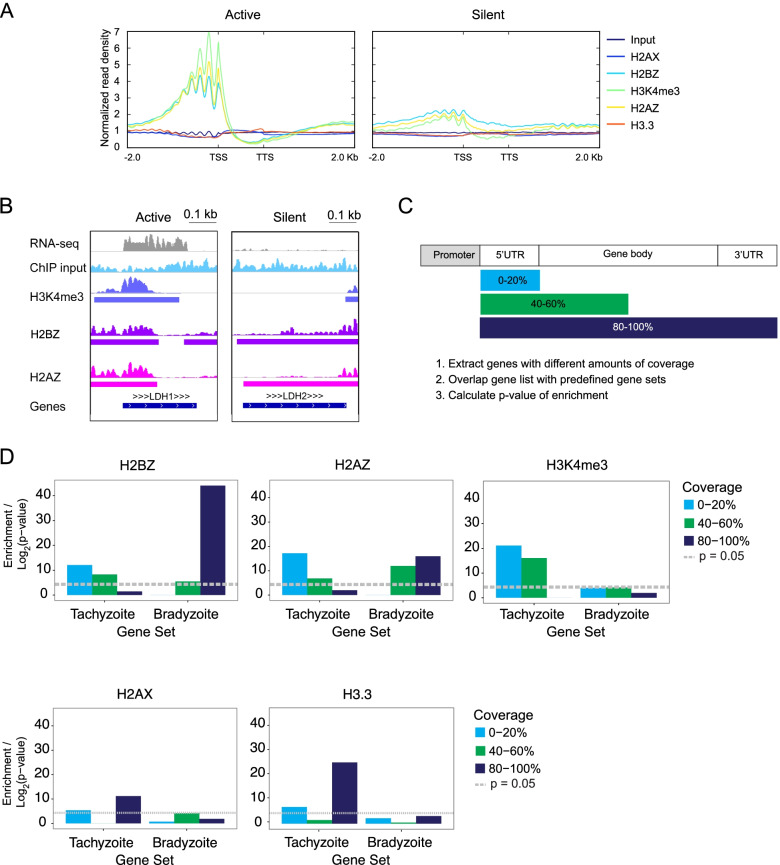
H2A.Z and H2B.Z mark active and silent genes. **A**. Average histone variant profiles at genes that are active or silent in tachyzoites stages based on RNA-seq data [31]. TSS (transcriptional start site) and TTS (transcriptional termination site) denote boundaries of genes, with 2 kb upstream and downstream flanking sequence. Gene boundaries as defined by genome annotation were used if experimental TSS and TTS were not available. ChIP-seq signals were normalized by scaling to library size. **B**. Genome browser view of H2A.Z, H2B.Z and H3K4me3 at an active (left panel) and a silent (right panel) gene. Peaks called using MACS2 are shown under read density profiles. **C**. Statistical approach for investigating link between histone variant gene coverage and stage-specific gene expression. Genes marked by histone variant ChIP peaks were split into groups depending on what percentage of the gene (starting at 5’UTR, to 3’UTR) is covered by a peak. Each group of genes was compared to predefined gene sets containing genes that are upregulated in different parasite stages [12, 29] and a measure of enrichment (*p*-value) was calculated using a hypergeometric distribution test. **D**. Plots showing -log2(*p*-value) of enrichment for genes with different amounts of H2A.Z, H2B.Z or H3K4me3 coverage compared to tachyzoite or bradyzoite gene sets. Genes with different levels of H2A.X or H3.3 coverage in tachyzoite or bradyzoites gene sets and significance of enrichment (−log_2_(*p*-value)) calculated are shown for comparison. *p*-value of < 0.05 in the hypergeometric test was considered significant and is indicated by the dotted grey line

Because H2A.Z also has a role in transcriptional repression [32], we examined its distribution on silent genes. Surprisingly, manual inspection of ChIP profiles suggested that in addition to localizing to active promoters, H2A.Z and H2B.Z are also present at low levels on gene bodies of silent genes, between TSS and transcription termination sites (TTS) (Fig. 5). We examined the genome-wide distribution of H2A.Z and H2B.Z on silent genes and stage-specific genes (Fig. 5A, additional file 6). Enolase 1, which is expressed in bradyzoites, and enolase 2, which is expressed in tachyzoites, map to a tandem array on chromosome VIII (Fig. 2A). Both H2A.Z and H2B.Z are observed on promoters of active *ENO2* gene but also on the top of the CDS of silent *ENO1* gene (Fig. 2A). Similarly, H2B.Z and H2A.Z are distributed over the CDS in silent *LDH2* gene and at the promoter of the active *LDH1* gene (Fig. 5 B and additional file 2).

To investigate whether this trend occurs genome-wide, we extracted lists of genes marked by histone variant peaks and performed gene set enrichment analysis using previously defined gene sets [21, 33]. This analysis was described previously to analyze *T. gondii* data and is commonly used for pathway analysis in numerous organisms. The *T. gondii* gene sets that were used were comprised of genes that are upregulated in different developmental stages, cell cycle time points or subcellular localization. A hypergeometric distribution test (also known as Fisher’s Exact Test) was used to calculate the statistical likelihood of enrichment or overrepresentation of genes in a particular gene set compared to a randomized gene set of the same size, yielding a *p*-value of enrichment which we term ‘enrichment score’. Overrepresented gene sets were defined as those with a *p*-value of < 0.05 in the hypergeometric test [21, 34, 35]. To test whether genes with different amounts of H2A.Z or H2B.Z coverage were overrepresented in tachyzoite or bradyzoite genesets, we split tachyzoite and bradyzoite genes into groups based on the percentage of the gene that was covered by an H2A.Z or H2B.Z peak (e.g. 0–20%, 20–40% of the gene length beginning at the promoter) coverage (Fig. 5C and D). Enrichment scores were plotted for histone variant peaks across genes expressed in both developmental stages (Fig. 5 D and additional file 6). While genes with lower H2A.Z and H2B.Z gene coverage (e.g. 0–20%) are statistically enriched in genes upregulated in tachyzoites, bradyzoite genes are overrepresented in high coverage gene sets (e.g. 80–100% coverage).

As a comparison, the same analysis was performed on H3K4me3, H2A.X and H3.3 peaks. As expected, since H3K4me3 is exclusively found at promoters in every organism to date, tachyzoites genes are overrepresented among genes with 0–40% H3K4me3 coverage, and genes with 40–60% H3K4me3 coverage are enriched in tachyzoite and not bradyzoites genes. Genes marked with 80–100% H2A.X and H3.3 are statistically enriched for tachyzoite genes, but not bradyzoite genes (Fig. 5D). Together, these findings suggest that the genome positions of H2A.Z and H2B.Z are linked to control of stage-regulated genes.

### Cell cycle control and histone variants

Canonical histones play a critical role in cell cycle control. Their synthesis is coupled to DNA replication during S-phase and is essential for chromatin assembly. In contrast, histone variants are synthesized throughout the cell cycle and whether they affect cell cycle progression is unclear [30]. To investigate whether histone variants play a role in regulating cell cycle dependent transcripts in *T. gondii*, we used the enrichment analysis method described above to compare genes with different amounts of histone variant coverage to cell cycle gene sets defined previously [33] . During the 8 h *T. gondii* cell cycle, there are two waves of transcription corresponding to G1 and S/M phases [36]. G1-regulated genes mainly have basal eukaryotic functions such as metabolism or biosynthetic processes, while SM-regulated genes are important for parasite maturation, division and biogenesis of parasite-specific organelles.

**Fig. 6:**
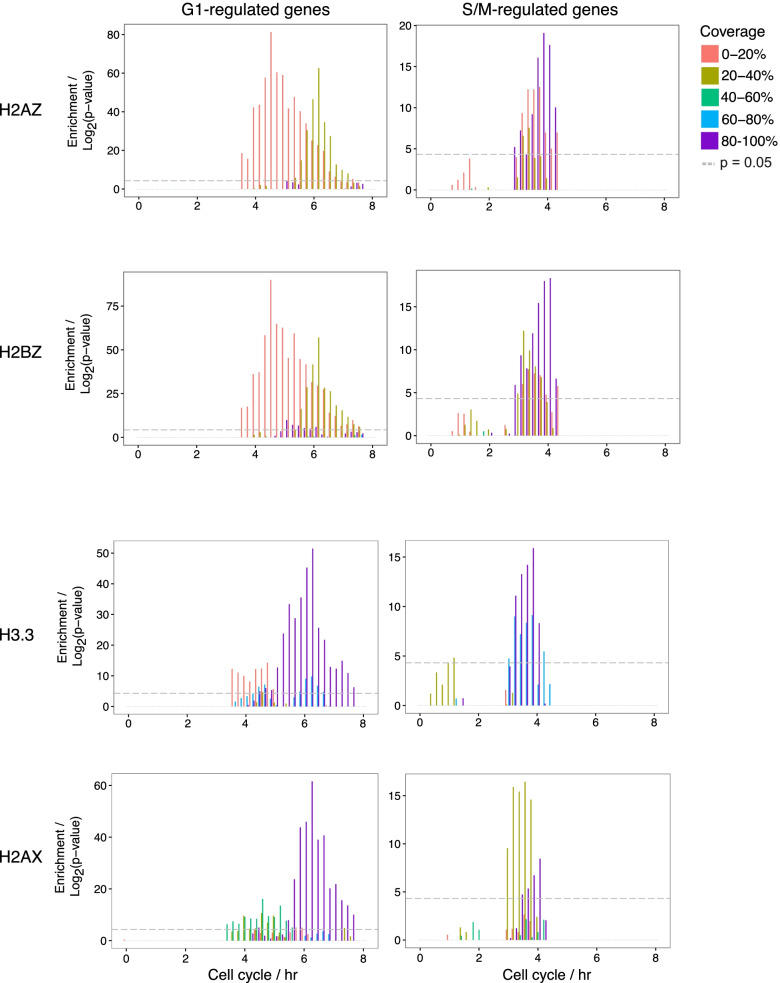
H2A.Z, H2B.Z, H3.3 and H2A.X distribution and gene coverage vary with gene expression within the cell cycle. Using the same strategy as shown in Fig. 5, genes with different amounts of H2A.Z, H2B.Z, H3.3 or H2A.X coverage were compared to sets of genes upregulated at intervals during cell cycle. Plots show -log_2_(*p*-value) of enrichment for genes with different levels of histone variant coverage. Genes with 0–20% H2A.Z or H2B.Z coverage are enriched in G1-regulated genes upregulated between hours 4 and 6 of cell cycle, while genes with 20–40% coverage are enriched in G1-regulated genes upregulated between hours 5 and 7. The pattern of histone variant H2A.Z, H2B.Z coverage differs in SM-regulated genes with enrichment of genes with significant coverage over the entire gene. Note that the enrichment scales for G1 and S/M are different. *p*-value of < 0.05 in the hypergeometric test was considered significant and is indicated by the dotted grey line

Genes with low amounts of H2A.Z and H2B.Z coverage (0–20% and 20–40%) are enriched in G1 genes, while genes expressed later in the cell cycle have higher amounts of coverage (Fig. 6). Interestingly, genes with 0–20% coverage are enriched in genes upregulated at a slightly earlier time point (4–6 h) than genes with 20–40% coverage (5–7 h), suggesting that coverage correlates with the timing of expression. Recent work using single cell sequencing [37] demonstrated that *T. gondii* G1 is divided into G1a followed by G1b, substages of G1 that are characterized by different sets of cell cycle expressed genes. Differentiated bradyzoites are predominantly in G1 [38] and single cell gene expression patterns of bradyzoites are those of G1b cells [37].

Turning to S/M-regulated genes, genes with both low and high levels of H2A.Z and H2B.Z coverage are overrepresented in genes upregulated in mid-S/M (3–5 h) phase. This is the time period during which parasites begin to express bradyzoite markers that differentiate to bradyzoites [38], and many parasite-specific proteins are upregulated at this point. Of note, a similar enrichment pattern of both high and low levels of H2A.Z and H2B.Z coverage were seen in merozoite genes, which are expressed in the feline intestine stages (additional file 6).

A similar analysis showed that genes with high coverage (80–100%) of H2A.X and H3.3 are enriched in G1 genes upregulated between 5 and 7 h of the cell cycle (Fig. 6). Genes with high H3.3 coverage were also enriched for mid-S/M-regulated genes, as well as genes with 20–40% H2AX. G1-regulated genes had higher H2AX and H3.3 coverage, consistent with the observation by manual inspection that these variants are present on gene bodies. Comparison of the genome-wide distributions of these variants suggests that the composition of histones within nucleosomes is dynamic and varies throughout the cell cycle as different sets of genes are transcribed.

## Discussion

*T. gondii*, an opportunistic parasite with worldwide distribution, has several developmental transitions that are regulated by epigenetic processes. Here, we provide a genome-wide overview of localization of *T. gondii* histone variants, which are linked to chromatin structure and regulation of gene expression. *T. gondii* has five histone variants: H3.3, H2A.X, H2A.Z, H2B.Z and CenH3. CenH3 has a conserved function at centromeric regions in eukaryotes including *T. gondii* [13] and we confirmed the localization of CenH3 to centromeres by ChIP-seq. To analyze the location of other variants across the genome, we used high-throughput chromatin immunoprecipitation followed by either microarray hybridization [22] or sequencing.

In tachyzoite stages, H3K4me3 is absent on promoters of bradyzoite genes and other silent genes. In the Apicomplexan *Plasmodium falciparum,* H3K4me3 and H3K9ac were shown to be present on promoters of active genes in schizont stage but on both active and silent genes in ring stages [39]. The difference in results may reflect divergent chromatin biology or may be a result of experimental conditions. *Plasmodium* varies from *T. gondii* in having a longer 48-h asexual cell cycle (vs 6–8 h for *T. gondii*), therefore chromatin state and gene expression can be examined simultaneously in synchronized cells, an approach that is technically challenging given the short life-cycle of *T. gondii* and the large amounts of biological material required for N-ChIP.

At *T. gondii* promoters, H3K4me3 colocalizes with H2A.Z and H2B.Z, whereas, when present on gene bodies, H2A.Z and H2B.Z are not accompanied by H3K4me3, suggesting an additional role for these histone variants, perhaps in gene silencing. An open chromatin state, marked by H2A.Z/H2B.Z, H4ac and H3K4me3, is required for expression of genes in asexual stages of both *Plasmodium* spp. and *T. gondii* [8, 40]. H2A.Z and H2B.Z form a dimer [14] and colocalize at promoter regions of active genes.

H2A.Z is the most evolutionarily conserved histone variant, while H2B.Z is specific to Apicomplexan parasites. Both these variants differ from canonical histones within the N-terminal domain. H2A.Z has an additional ~ 20 aa region that is extensively modified by PTM [20]. In *P. falciparum,* the H2B.Z/H2A.Z dimer is enriched at TSS and 5′-UTR and is associated with well-known activation marks H3K4me3 and H3K9ac as well as H3K18ac and H3K27ac [41]. The H2A.Z and H2B.Z dimer is excluded from heterochromatin regions such as silent *var* genes encoding variant antigens, but is present at the promoter of the single active *var* copy [25].

Bradyzoite genes are silent in tachyzoites and rarely expressed in the RH strain. H2A.Z/H2B.Z coverage of silent bradyzoite genes extends from the promoter along the gene body, but the levels detected are lower than the prominent peaks seen at promoters of active tachyzoite genes. We also observed enrichment of H2A.Z and H2B.Z on gene bodies on a subset of merozoite genes (additional file 6). We hypothesize that genes with H2A.Z/H2B.Z coverage extending along gene bodies are poised for transcription, as occurs in yeast [42, 43] and embryonic stem cells [44]. In mammalian embryonic stem cells, poised but silent promoters are marked by H3K4me3 and H3K27me3 as well as H2A.Z [44]. In *T. gondii*, these broad H2A.Z/H2B.Z peaks may play a role in state-specific gene regulation during the tachyzoite-bradyzoite transition.

It is likely that differences in PTM of H2A.Z/H2B.Z or other histones within the same nucleosome are responsible for regulating transcription positively or negatively. In cancer cells, acetylated H2A.Z (H2A.Zac) is enriched at promoters of poised and active genes, but deacetylated H2A.Z [45] is spread throughout the entire promoter when the gene is silent. In *T. gondii*, H2A.Z and H2B.Z are extensively acetylated in the N-terminal domain [20], a classic mark of transcriptional activation. In contrast, relatively few acetylation sites have been detected on canonical H2A, H2B or H2A.X in tachyzoites [20]. Taken together with the current study, it is likely that acetylated H2A.Z and H2B.Z localize to promoter regions and play a role in transcriptional regulation. Future studies in *T. gondii* examining the distribution of H2A.Zac/H2B.Zac and deacetylated forms are needed, to confirm whether the acetylation state of H2A.Z/H2B.Z is necessary for transcriptional activation [46].

Several other PTM have been detected on H2A.Z [47, 48]. While hyperacetylated H2A.Z is associated with transcriptional activation, ubiquitinated or methylated H2A.Z is associated with gene repression [32]. In *T. gondii*, H2A.Z and H2B.Z each have seven ubiquitination sites in both N- and C-terminal domains with unidentified functions [21]. Ubiquitination was detected on TgH2AK119Ub and TgH2BK120Ub [21], residues that are important for recruitment of repressive complexes [49] and transcriptional activation [50], respectively. H2A K119 ubiquitination is mediated by E3 ligase activity of the polycomb repressive complex PRC1 [51]. Acetylation and ubiquitination may regulate the differential localization of H2A.Z and H2B.Z on active and silent genes in *T. gondii*. Acetylated H4K31 is enriched in promoters of active genes, whereas methylated H4K31 is found in silent regions and the body of inactive genes [29]. Collectively, these data suggest that coordinated deposition of other PTM on histones plays a critical role in *T. gondii* gene expression and developmental transitions.

In mammalian cells, deacetylated H2A.Z localizes to centromeres and co-localizes with the classic centromeric marks H3K4me2, H3K9me2 or H3K9me3 [52, 53]. In addition, H2A.Z associates with subtelometric regions and other heterochromatin boundaries, where it suppresses the spread of heterochromatin caused by Sir2 deacetylase activity [54]. H2A.Z/H2B.Z were not enriched at centromeric or telomeric-TAS regions in our study, suggesting that these variants have little or no role controlling heterochromatin architecture in *T. gondii*.

H2A.X is commonly associated with double strand DNA break repair, including in *T. gondii* [14], but also functions in transcriptional silencing, chromosome segregation, and senescence [55]. H2A.X localizes to different loci than H2A.Z/H2B.Z, and is enriched at the chromosome ends and centromeres, together with H3.3 [11]. In contrast to the literature, our genome-wide study shows that repressed loci are not significantly enriched with H2A.X, and instead, genes transcribed in tachyzoites are marked by H2A.X within gene bodies.

**Fig. 7:**
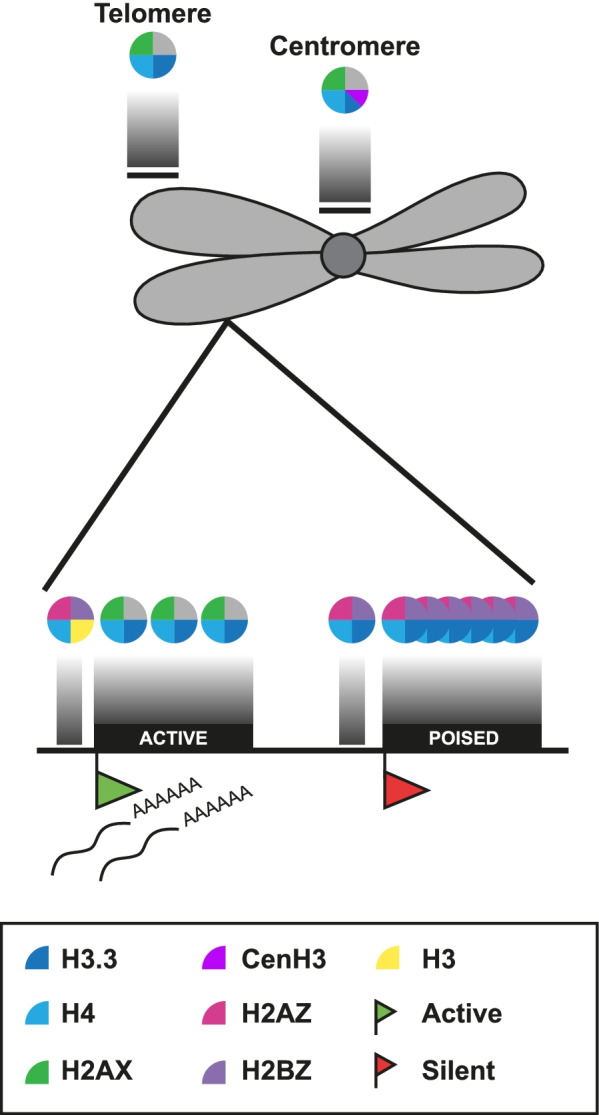
Model of stage-specific gene regulation by histone variants. Model of different nucleosomal composition on *T. gondii* chromatin. Different chromatin regions have distinct configurations that differentially change the chromatin architecture according to DNA-related processes. Nucleosomes in typical heterochromatin regions such as centromeres contain the specific centromeric histone CenH3, together with H2A.X. H3.3 enrichment is also evident in those regions, suggesting an atypical heterodimer with CenH3 and H3.3 or alternate CenH3 and H3.3-containing nucleosomes. H2A.X likely forms a tetramer with an unknown H2B, probably H2Ba. Canonical H2Ba with H2A.X is located at opposite sites to H2A.Z/H2B.Z dimer. H3.3 and H2A.X are also enriched at chromosome ends. Transcription start sites (TSS) of active genes are enriched with the variant dimer H2A.Z/H2B.Z. On the CDS of these genes, H2A.Z/H2B.Z are replaced with H2A.X (with H2Ba), and the canonical histone H3 by H3.3. Transcriptionally silenced genes have a similar composition with H2A.Z, H2B.Z and H3.3 at the TSS. But this configuration extends throughout the CDS of these genes, especially at genes which are specific for bradyzoites. These regions do not contain H3K4me3, a landmark for active transcription, suggesting that these genes could be poised. H4 has no variants, so it should be present throughout the genome

Functional regions of chromatin, telomeres and centromeres, have distinct structures and composition. This study provides insight into the composition of nucleosomes present in these regions in *T. gondii* (Fig. 7)*.* In eukaryotes, CEN-PA (homologue of CenH3) replaces canonical H3 in centromeric chromatin [56]. In *T. gondii,* H3.3 and CenH3 both appear to be present in centromeric regions, as confirmed by mass spectrometry (unpublished data). In contrast to *T. gondii, P. falciparum* H3.3 is not present at centromeric regions [57], suggestive of different chromatin regulatory mechanisms in these two Apicomplexa species. Our findings mirror those in *Drosophila melanogaster* and humans, where centromeric chromatin was shown to contain CEN-PA containing nucleosomes interspersed with canonical H3-containing nucleosomes [58].

In mammalian cells, CEN-PA is recruited to centromeric regions by formation of double stranded breaks to which H2A.X is also recruited [59]. Given the localization of H2A.X at centromeric regions, this mechanism may be conserved in *T. gondii.* In addition, H3.3 and H2A.X were both detected at telomeric regions in *T. gondii*. In mammalian cells, H2A.X is important for the correct arrangement of telomere ends during meiotic prophase [60], while H3.3 is deposited at telomeric regions where it is important for facilitating heterochromatin assembly [61].

As well as histone variants having a potential role in stage-specific gene expression, we also noted a cell cycle-dependent pattern of variant histone distribution. In these experiments with asynchronous cultures, which are primarily in G1 phase, genes with less H2A.Z/H2B.Z coverage (covering only the 5′ end of the gene) are those upregulated at an earlier time point of cell cycle compared to those with more coverage. Genes where the H2A.Z/H2B.Z dimer colocalizes with H3K4me3 on promoters and also localizes on gene bodies, are genes expressed in mid-S/M phase. The timing of the tachyzoite-bradyzoite developmental transition is also linked to the cell cycle [37, 38, 62], so distribution and PTM modifications of the H2A.Z/H2B.Z dimer could provide a mechanism by which *T. gondii* integrates signals to either initiate the differentiation program or begin DNA synthesis and cell replication.

## Conclusions

Together, this work suggests that histone variants regulate cell cycle and stage-specific gene expression in *T. gondii*. We hypothesize that variant histones regulate stage-specific gene expression by demarcating functional regions of chromatin. H2A.Z/H2B.Z dimer is implicated as a critical player in regulation of both gene activation and gene silencing. Future work is needed to determine how the localization of histone variants evolves during *T. gondii* bradyzoite differentiation and the mechanisms by which H2A.Z/H2B.Z and H2A.X/H2B dimers are exchanged.

## Methods

### Cell culture and parasite infection

HFF cells (Human Foreskin Fibroblasts were originally isolated from pooled human foreskins at Stanford University in 1994 and preserved as low passage primary cell line stocks) were grown in Dulbecco’s Modified Eagle Medium (DMEM), supplemented with 10% fetal bovine serum, 2 mM L-glutamine, 100 U/ml penicillin and 100 μg/ml streptomycin and maintained at 37 °C with 5% CO_2_. Confluent HFF cells were infected with tachyzoite forms of *T. gondii* (strain Type I RH*Δhxgprt* [63]) at a ratio of 3–5 parasites per cell. At around 40 h post infection, cells were harvested and lysed sequentially with 20G/23G/25G needles.

Endogenous epitope tagged variant H2A.X histone strain in the Type I RH*Δhxgprt* background [14] was subjected to chromatin immunoprecipitation (IP) without previous purification from host cell debris. The H2B.Z-myc strain was generated by cloning the *H2B.Z* ORF in the plasmid pTUB8mycGFPtailTy-HX in frame with a c-Myc tag at the N-terminal end using selection for *HXPRT* into the RH*Δhxgprt* strain. The H3.3-HA was cloned by placing the *H3.3* ORF in-frame with a C-terminal HA tag and was also transfected into the Type I RH*Δhxgprt* background, and stable clones created using selection for *HXPRT*. For IPs that required antibodies, parasites were purified from host cell debris using a 3.0 μm Nucleopore filter (Whatman) until no human cells were visualized. After two washes with cold PBS, cell pellets were frozen in liquid nitrogen.

### Indirect immunofluorescence

Indirect immunofluorescence experiments were performed on HFF cells grown on coverslips until they reached confluence. Cells were infected with 10^6^ freshly lysed tachyzoites. After 24 h, samples were fixed with 4% *p*-formaldehyde in PBS for 20 min at room temperature (RT). Fixed cells were permeabilized with 0.25% of Triton X-100 for 10 min at RT followed by blocking with 1% of bovine serum albumin (BSA) for 30 min. Primary antibodies were diluted in PBS and incubated for 2 h at RT. For the tagged lines, the following commercial antibodies were used: H3.3-HA tag (Rat monoclonal antibody/ HA:1:500- Roche Cat No. 11867423001); H2A.X-myc and H2B.Z-myc (Rabbit polyclonal antibody/1:1000-Invitrogen Cat # 13–2500). We also used specific polyclonal antibodies against *T. gondii* histone variants H2A.Z, H2B.Z and H2A.X (1:500 [14];).

Following incubation, cells were washed twice with PBS and incubated with secondary antibodies (Alexa fluor 488-Molecular Probes Cat No A-11034) and 10 μg/mL of 4′,6-diamidino-2-phenylindole (DAPI) to detect nucleic acids. Slides were mounted with Vectashield (Vector Laboratories) and analyzed with a Delta Vision Elite Core Deconvolution microscope. Imaging was performed on an inverted Olympus IX71 microscope with 60x objective (Albert Einstein College of Medicine Analytical Imaging Facility); Z-stacks of 0.1 μm were collected and deconvolution was performed using SoftWoRx 3.6.0. Images were processed with ImageJ Fiji 1.46 J, Adobe Photoshop CS3 and Adobe Illustrator CS3.

### Native chromatin immunoprecipitation (N-ChIP)

Freshly collected or frozen parasites (3-5 × 10^8^) were resuspended in Suspension Solution (SS) (300 mM sucrose; 15 mM Tris-HCl pH 7.4; 5 mM MgCl_2_, 15 mM NaCl; 60 mM KCl; 0.1 mM EDTA; 0.1 mM PMSF; protease inhibitors) and lysed in the same solution with 0.2% NP-40 for 5 min on ice. Lysed parasites (nucleus-enriched) were washed once with SS and once with MNase Solution (0.32 M sucrose; 50 mM Tris-HCl pH 7.5; 4 mM MgCl_2_; 1 mM CaCl_2_; 0.1 mM PMSF and protease inhibitors). MNase (Micrococcal nuclease (USB) Cat # 70196Y) digestion was performed in 100 μl of the same solution and 5 U of the enzyme for 5 min at 37 °C. The reaction was stopped by adding 200 mM EDTA and incubating for 5 min on ice. After centrifuging, supernatants were stored (sample 1). Pellet were resuspended in 1 ml Lysis Buffer (1 mM Tris-HCl pH 7.5; 0.2 mM EDTA; 0.3 mM PMSF) and dialyze overnight with lysis buffer (Slide-A-Lyzer Dialysis Cassette 3.5 K MWCO, 3 mL, Cat. no. 66330 Pierce).

After dialysis, the supernatant was combined with sample 1 and diluted 10 fold in dilution buffer (0.01% SDS; 1.1% Triton X-100; 1.2 mM EDTA; 16.7 mM Tris-HCl; 167 mM NaCl and protease inhibitors; pH 8.1). The sample was precleared with magnetic beads for 30 min at 4 °C and incubated with 20 μl polyclonal antibodies or 2 μl anti-HA, Myc or H3K4me3 (Millipore cat No 07–473) overnight at 4 °C with rotation. DNA-protein-antibody complexes were recovered by magnetic beads coupled to Protein A (Thermo Fisher Cat No 10001D) for 2 h at 4 °C and beads were washed 3 times with the following solutions before washing 6 times with TE (Triton-EDTA) buffer: Low Salt Wash Buffer: 0.1% SDS, 1% Triton X-100, 2 mM EDTA, 20 mM Tris-HCl, 150 mM NaCl, pH 8.1; high salt wash buffer: 0.1% SDS, 1% Triton X-100, 2 mM EDTA, 20 mM Tris-HCl, 500 mM NaCl, pH 8.1; LiCl wash buffer: 0.25 M LiCl, 1% NP40, 1% deoxycholate, 1 mM EDTA, 10 mM Tris-HCl, pH 8.1. Elution was performed with freshly made elution buffer (1% SDS; 0.1 M NaHCO_3_) twice for 15 min each at room temperature with rotation. A sample of input DNA was saved before antibody incubation and the final eluted samples were treated with proteinase K for 1 h at 45 °C.

Eluted DNA was recovered using MinElute PCR Purification Kit, Cat. no. 28006, Qiagen). The concentration of the purified DNA was determined with the Quant-iT™ PicoGreen system (Invitrogen). ChIP samples were submitted to the Einstein Epigenomics Shared Facility (ESF) for sequencing. Adapters were ligated to DNA to create the Illumina library and sequenced using a Hiseq2500 sequencing machine (Illumina). This involves a first step of cluster generation in situ on the flow cell followed by massively-parallel sequencing using fluorescently-labeled reversible terminator nucleotides. Clustered templates were sequenced base-by-base during each read. Images generated by the Hiseq2500 were processed by Hiseq Sequence Control Software (HCS V2 with RTA v1.17 and CASAVA 1.8 software packages). These software components perform the functions of image analysis (Firecrest) and base-calling (Bustard).

### ChIP-seq data processing

Illumina adapter sequences were trimmed from reads using cutadapt (https://cutadapt.readthedocs.io/en/stable/) and aligned to the *T. gondii* ME49 genome (release 26, ToxoDB.org) using Bowtie2 (http://bowtie-bio.sourceforge.net/bowtie2/index.shtml). For coverage plots, aligned BAM files were normalized by library size and converted to bigwig format using deeptools bamCoverage (http://deeptools.readthedocs.io/en/).

### Comparison of ChIP-seq datasets at the read level

Bigwig files were compared by cluster analysis using deeptools computeMatrix (http://deeptools.readthedocs.io/en/latest/) to calculate Pearson correlations between the read densities of samples followed by visualization in a heat map generated using deeptools plotHeatmap (http://deeptools.readthedocs.io/en/latest/content/tools/plotHeatmap.html).

*ChIP-seq peak calling and comparisons.*

Peaks were called using MACS2 (https://github.com/taoliu/MACS) and Homer FindPeaks (http://homer.ucsd.edu/homer/). Peak lists were compared using the findOverlapsOfPeaks function in in the Bioconductor R package ‘ChIPpeakAnno’ (http://bioconductor.org/packages/release/bioc/html/ChIPpeakAnno.html). For histone variants where the enriched domains were generally less than 5000 bp, MACS2 was applied (H3K4me3, H2A.Z, H2B.Z); for proteins with enriched domains larger than 5000 bp (H2A.X, H3.3, CenH3) and with high similarity to input, Homer was used (http://homer.ucsd.edu/homer/motif/). A minimum FDR of 0.05 was applied.

### ChIP-chip data processing

Pair files (IP and input) were combined and processed using NimbleScan software (NimbleGen) according to the software manufacturer’s instructions. A minimum FDR of 0.05 was applied to define peaks. Scaled log transformed ratio files (IP/input) were generated in NimbleScan and visualized with IGV software.

#### Comparison of ChIP-seq and ChIP-chip data

Correlations between ChIP-seq and ChIP-chip were calculated at the peak level using the Bioconductor R package ‘diffbind’ (http://bioconductor.org/packages/release/bioc/html/DiffBind.html). Peak lengths and numbers were calculated from peak files determined from NimbleScan (ChIP-chip), MACS2 (ChIP-seq) and Homer (ChIP-seq).

#### Collective annotation of ChIP peaks

To obtain a list of reproducibly detected peaks that are common to all ChIP-seq and ChIP-chip experiments for H3K4me3 or histone variants, the findOverlapsOfPeaks function in the Bioconductor R package ‘ChIPpeakAnno’ (http://bioconductor.org/packages/release/bioc/html/ChIPpeakAnno.html) was used to generate a merged BED file containing all overlapping peaks with a minimum of 50 bp overlap. Peaks were annotated using ChIPpeakAnno annotatePeakInBatch function using a GFF genome annotation file downloaded from ToxoDB.org (*T. gondii* ME49, release 26 and limited to 1000 bp distance to a genomic feature. Plots of peak locations were generated using ggplot2.

Prior to downstream analysis, for each histone mark or variant, called peaks from biological replicates were intersected; only peaks that were present in all analyses were combined for assigning genes to peaks. The correlation plots in Fig. 2B were generated using raw ChIP-seq files, and the correlation plots in additional file 2C were generated using called peaks from ChIP-seq or ChIP-chip.

#### Average ChIP-seq profiles over genomic features

Deeptools plotProfile (http://deeptools.readthedocs.io/en/latest/index.html) was used to plot composite ChIP-seq profiles by averaging the read density of library size-normalized BigWig files over various genomic regions, with 500 bp upstream and downstream flanking regions.

### Enrichment analysis of genes with different histone variant coverage

Lists of genes where histone variants localize were analyzed by pathway and gene set enrichment analysis in R. Gene sets consisting of genes upregulated at different cell cycle time points, GO and KEGG pathways and localization [33] were compared to the histone variant gene lists by a hypergeometric test as previously described [21]. Briefly, *p*-values of enrichment were determined and adjusted for multiple hypotheses testing by the Bonferroni method. Adjusted *p*-values were divided by *p*-values generated by 1000 random iterations of each gene set, generating a normalized p-value used to generate plots. Scripts for enrichment analysis and annotation files can be found at https://github.com/nataliesilmon/toxotools. *p*-value of < 0.05 in the hypergeometric test were considered significant.

### Motif analysis

DNA sequences from overlapping H2A.Z, H2B.Z and H3K4me3 peaks were extracted and searched for motifs using the commandline version of Homer (http://homer.ucsd.edu/homer/motif/), compared to a FASTA file containing either the entire *T. gondii* genomic sequence or promoter regions as the background.

## Supplementary Information

**Additional file 1. **Summary of samples. **A.** Summary of samples and replicates of ChIP-chip and ChIP-seq experiments analyzed in this study. **B.** Data analysis workflow. ChIP-chip and ChIP-seq data were processed independently and combined at the peak level. ChIP-chip data were processed in the program NimbleScan to generate a ratio file (experiment/ input signal) and IGV format file for visualization. The ratio file was used to determine statistically significant peaks of ChIP-chip signal enrichment using NimbleScan. ChIP-seq data was preprocessed by trimming adapter sequences from raw reads and assessing quality using FASTQC. Reads were mapped to the *T. gondii* genome and converted to BigWig format for visualization and downstream analysis. Statistically significant peaks were identified using either the program MACS2, using parameters for detection of narrow or broad peaks, or the program Homer, with or without an input file as a control. Correlations between BAM files were determined and graphically represented using the function computeMatrix from the package deeptools. Read density plots were generated from BigWig files. Correlations between statistically significant (FDR < 10%) ChIP-seq and ChIP-chip peaks were calculated and a clustered heat map was generated using deeptools. Peaks detected using both platforms and peaks detected in replicates were intersected to provide a set of reproducible peaks for each histone variant. Reproducible peaks were annotated to determine closest genomic features including genes. Genes where histone variants were localized to were subjected to enrichment analysis to determine whether they are enriched for genes with particular function, localization or specific timing of expression. In addition, DNA sequences corresponding to reproducible peaks were analyzed to determine whether they contained motifs that could be bound by transcription factors.

**Additional file 2. **Correlations between ChIP-chip and ChIP-seq datasets. **A.** Genome browser view of ChIP-chip ratios over various genes in *T. gondii.* ChIP-chip outputs plot the scaled log-transformed ratio of experimental IP to input. Genes are identical to those displayed in Fig. 2A. **B.** Plot showing number of peaks detected using ChIP-seq and ChIP-chip platforms. The mean number of peaks detected in replicates are shown. **C.** Correlations between all peaks detected by ChIP-seq or ChIP-chip were calculated from peaks called with Homer (H2AX, H3.3 ChIP-seq), NimbleScan (all ChIP-chip) or MACS (all the others). Data was analyzed and plotted using the R package Diffbind. Color bar above the heat map indicates factor (histone variant – see heat map labels), technique (ChIP-seq – blue, ChIP-chip - pink), and replicate (purple = 1; pink = 2; blue = 3). The heat map color key is shown in the upper left with darker blue indicating the strongest correlations. As shown in the heat map, the H2A.Z, H2B.Z and H3K4m3 peaks from ChIP-chip and ChIP-seq showed the strongest correlations. Peaks for each variant or histone modification are the intersection of peaks from each biological replicate (ChIP-seq and ChIP-chip). **D.** Plot showing lengths of peaks from ChIP-seq and ChIP-chip platforms. Data from replicates were combined into one sample.

**Additional file 3. **Correlation between ChIP-seq datasets. Clustered heat map of correlations between all ChIP-seq alignment files for: **A.** 1 kb bins genome-wide; **B.** Restricted to promoter regions only. Samples are indicated on the Axes (see additional file 1 for list) with IP indicating ChIP and In indicating input sample for each experimental sample. Color key indicating correlation coefficient (Spearman) indicated below (negative correlation in blue, positive correlation in red).

**Additional file 4. **Distribution of histone variant ChIP-seq peaks over genes. **A.** Percentage of all annotated genes marked by histone variant peaks (regardless of location of peaks) is plotted. **B, C, D.** Plots depicting the percentage of ChIP-seq peaks with different locations of ChIP-seq peaks relative to the annotated promoter (1 kb upstream of transcriptional start site) (**B**), transcriptional start site (TSS) (**C**), gene start codon (**D**).

**Additional file 5. **Average histone variant ChIP-seq profiles across different genomic features. Plots show normalized read density (experimental IP/input) for each ChIP-seq sample. **A.** RNA gene classes include: transcribed genes mRNA, ribosomal RNA rRNA, transfer RNA tRNA. **B.** Genes with different mRNA expression levels in tachyzoites. *T. gondii* genes were split into 5 equal groups based on their expression level in tachyzoites (determined from RNA-seq of *T. gondii* RH strain tachyzoites [31]). Average normalized ChIP-seq read densities were calculated over genes in each group and plotted. Lightest grey indicates highest quintile expression, whereas black indicates genes that had no detectable expression. **C.** G1- and S/M- regulated genes. *T. gondii* genes were previously classified those that are transcribed in accordance with G1 or S/M phase of cell cycle [29]. Average normalized ChIP-seq read densities were calculated for genes in each set and plotted.

**Additional file 6. **Stage-specific gene regulatory patterns of histone variants. Genes with different amounts of histone variant coverage were compared to sets of genes upregulated in different parasite life cycle stages (defined in [21, 29]. –log_2_(*p*-value) of enrichment is plotted for genes with different levels of coverage for H2AZ and H2BZ. *p*-value of < 0.05 in the hypergeometric test was considered significant and is indicated by the dotted grey line.

## Data Availability

Sequencing and microarray data have been deposited at NCBI GEO, accession numbers GSE87834, GSE104347.

## Abbreviations

ChIP: Chromatin immunoprecipitation
N-ChIP: Native chromatin immunoprecipitation
ChIP-seq: Chromatin immunoprecipitation followed by high throughput sequencing
ChIP-chip: Chromatin immunoprecipitation followed by microarray hybridization
IP: Immunoprecipitation
H2A: Histone 2A
H3: Histone 3
H2B: Histone 2B
H4: Histone 4
CenH3: Centromeric histone 3 variant
UTR: Untranslated region of mRNA
TSS: Transcription start site
TTS: Transcription termination site
PTM: Post translational modifications
PCR: Polymerase chain reaction
MN: Micrococcal nuclease
