# Supplementary material for: Genome-wide localization of histone variants in Toxoplasma gondii implicates variant exchange in stage-specific gene expression
Source: BMC Genomics. 2022 Feb 14;23:128. doi: 10.1186/s12864-022-08338-6 (PMC8842566; doi:10.1186/s12864-022-08338-6)

Additional file 1

A

	Replicates	
**Sample**	**ChIP-seq**	**ChIP-chip**
H3K4me3	2	1
H2AZ	2	2
H2AX	2	2
H2BZ	3	None
H3.3	2	None
H4	1	None
CenH3	1	1
Input	13	(no independent profiles)

B

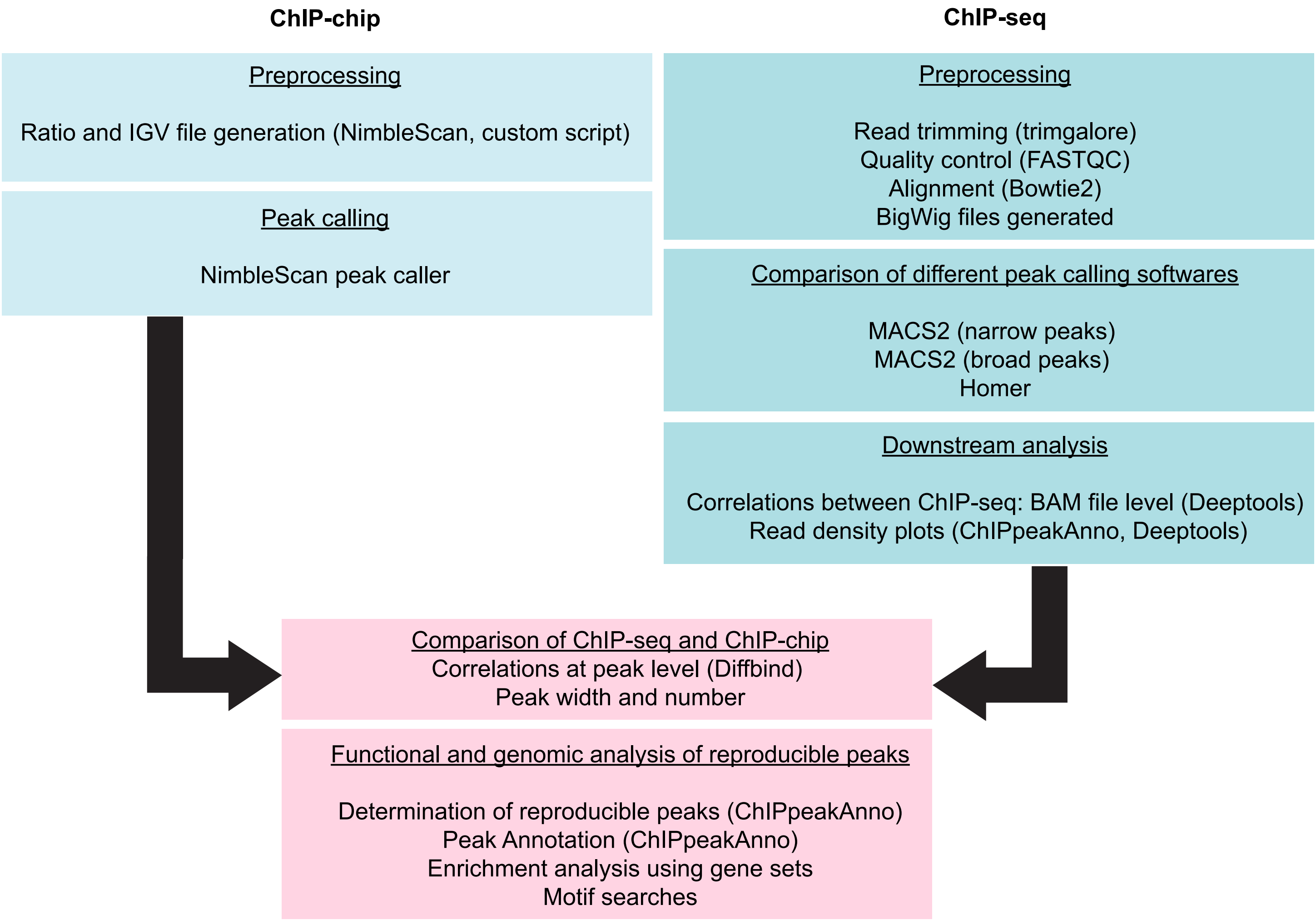

Supplement: Supplementary file 1 — Additional file 1. Summary of samples. A. Summary of samples and replicates of ChIP-chip and ChIP-seq experiments analyzed in this study. B. Data analysis workflow. ChIP-chip and ChIP-seq data were processed independently and combined at the peak level. ChIP-chip data were processed in the program NimbleScan to generate a ratio file (experiment/ input signal) and IGV format file for visualization. The ratio file was used to determine statistically significant peaks of ChIP-chip signal enrichment using NimbleScan. ChIP-seq data was preprocessed by trimming adapter sequences from raw reads and assessing quality using FASTQC. Reads were mapped to the T. gondii genome and converted to BigWig format for visualization and downstream analysis. Statistically significant peaks were identified using either the program MACS2, using parameters for detection of narrow or broad peaks, or the program Homer, with or without an input file as a control. Correlations between BAM files were determined and graphically represented using the function computeMatrix from the package deeptools. Read density plots were generated from BigWig files. Correlations between statistically significant (FDR < 10%) ChIP-seq and ChIP-chip peaks were calculated and a clustered heat map was generated using deeptools. Peaks detected using both platforms and peaks detected in replicates were intersected to provide a set of reproducible peaks for each histone variant. Reproducible peaks were annotated to determine closest genomic features including genes. Genes where histone variants were localized to were subjected to enrichment analysis to determine whether they are enriched for genes with particular function, localization or specific timing of expression. In addition, DNA sequences corresponding to reproducible peaks were analyzed to determine whether they contained motifs that could be bound by transcription factors. [file 12864_2022_8338_MOESM1_ESM.pdf]
