# Supplementary figures and images for: Genome-wide localization of histone variants in Toxoplasma gondii implicates variant exchange in stage-specific gene expression
Source: BMC Genomics. 2022 Feb 14;23:128. doi: 10.1186/s12864-022-08338-6 (PMC8842566; doi:10.1186/s12864-022-08338-6)

A

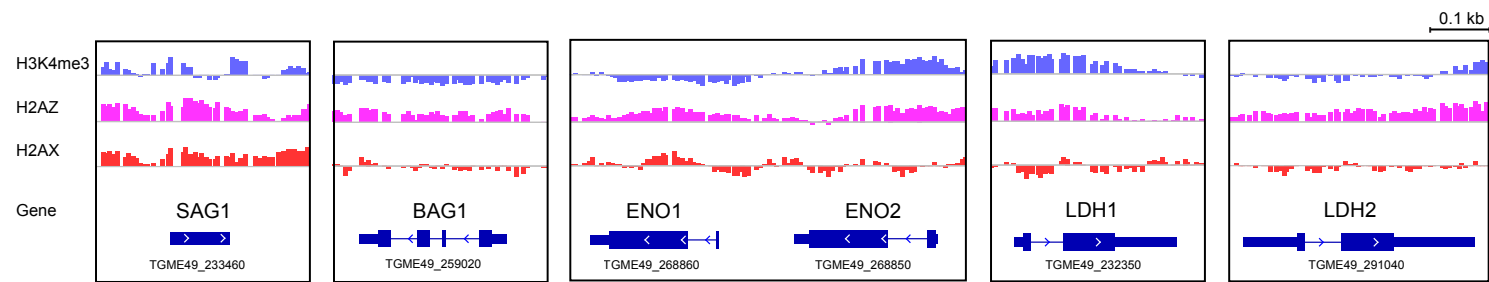

B

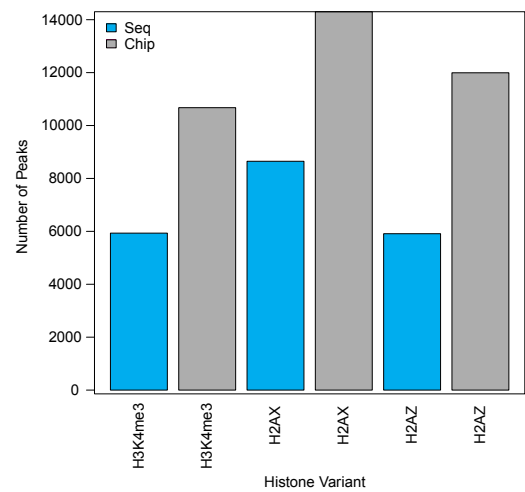

C

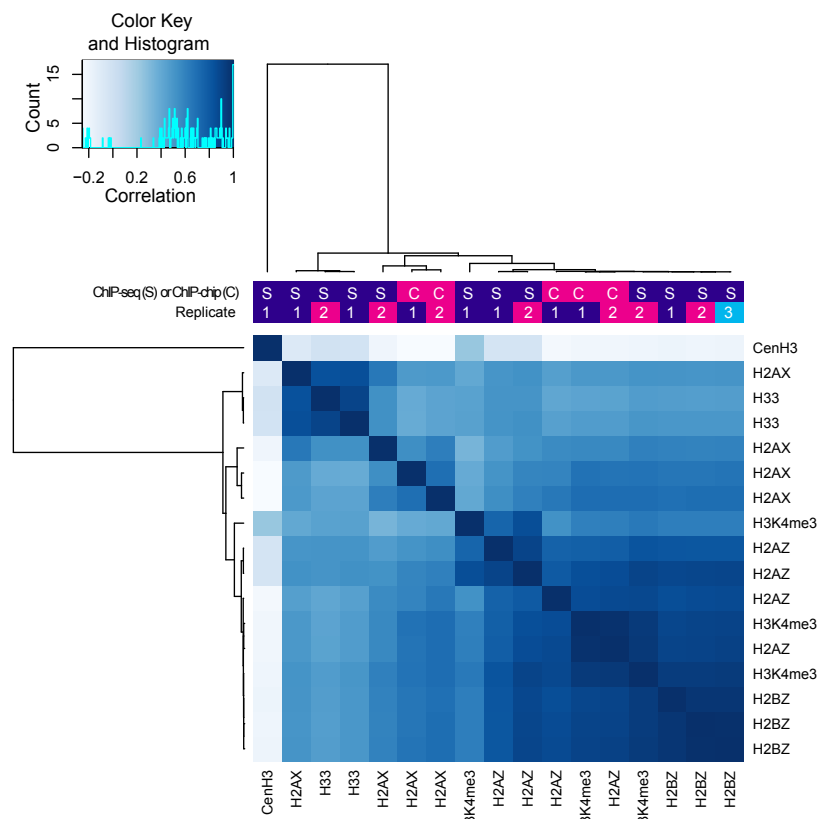

D

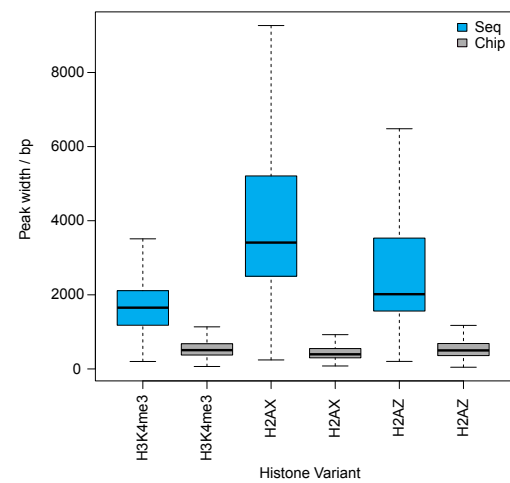

Supplement: Supplementary file 2 — Additional file 2. Correlations between ChIP-chip and ChIP-seq datasets. A. Genome browser view of ChIP-chip ratios over various genes in T. gondii. ChIP-chip outputs plot the scaled log-transformed ratio of experimental IP to input. Genes are identical to those displayed in Fig. 2A. B. Plot showing number of peaks detected using ChIP-seq and ChIP-chip platforms. The mean number of peaks detected in replicates are shown. C. Correlations between all peaks detected by ChIP-seq or ChIP-chip were calculated from peaks called with Homer (H2AX, H3.3 ChIP-seq), NimbleScan (all ChIP-chip) or MACS (all the others). Data was analyzed and plotted using the R package Diffbind. Color bar above the heat map indicates factor (histone variant – see heat map labels), technique (ChIP-seq – blue, ChIP-chip - pink), and replicate (purple = 1; pink = 2; blue = 3). The heat map color key is shown in the upper left with darker blue indicating the strongest correlations. As shown in the heat map, the H2A.Z, H2B.Z and H3K4m3 peaks from ChIP-chip and ChIP-seq showed the strongest correlations. Peaks for each variant or histone modification are the intersection of peaks from each biological replicate (ChIP-seq and ChIP-chip). D. Plot showing lengths of peaks from ChIP-seq and ChIP-chip platforms. Data from replicates were combined into one sample. [file 12864_2022_8338_MOESM2_ESM.pdf]

A

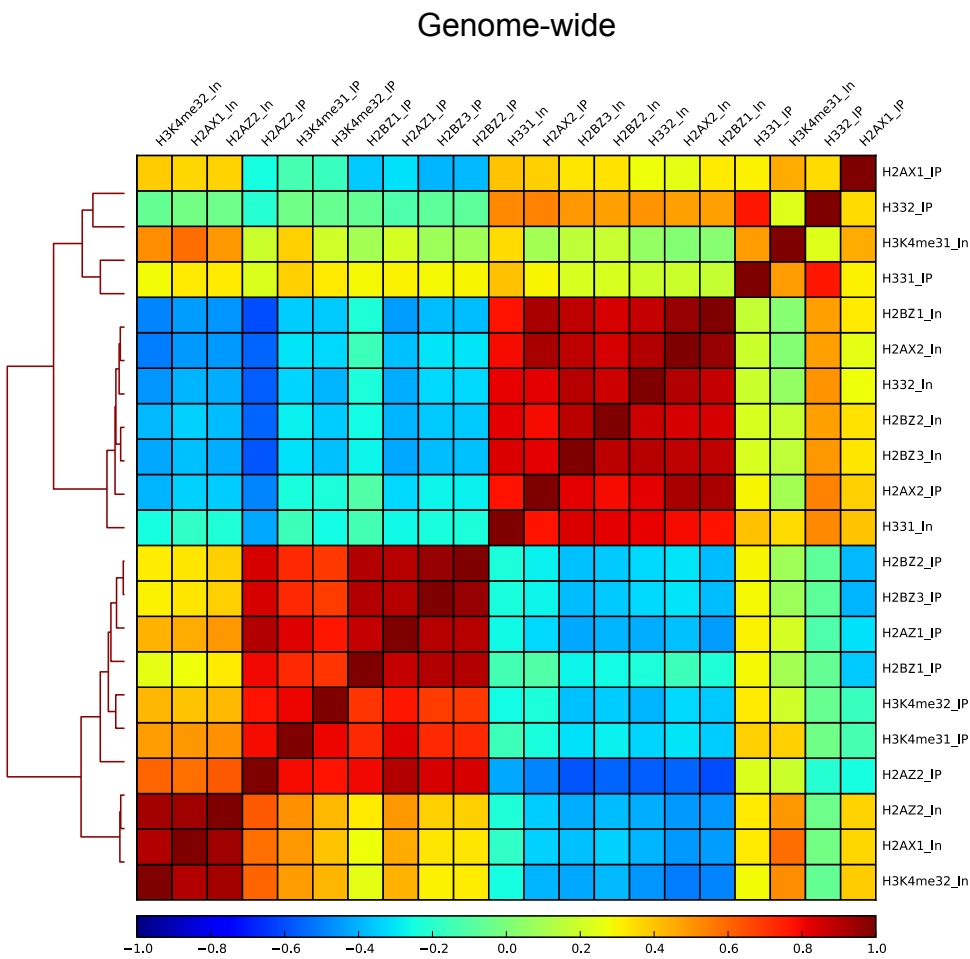

B

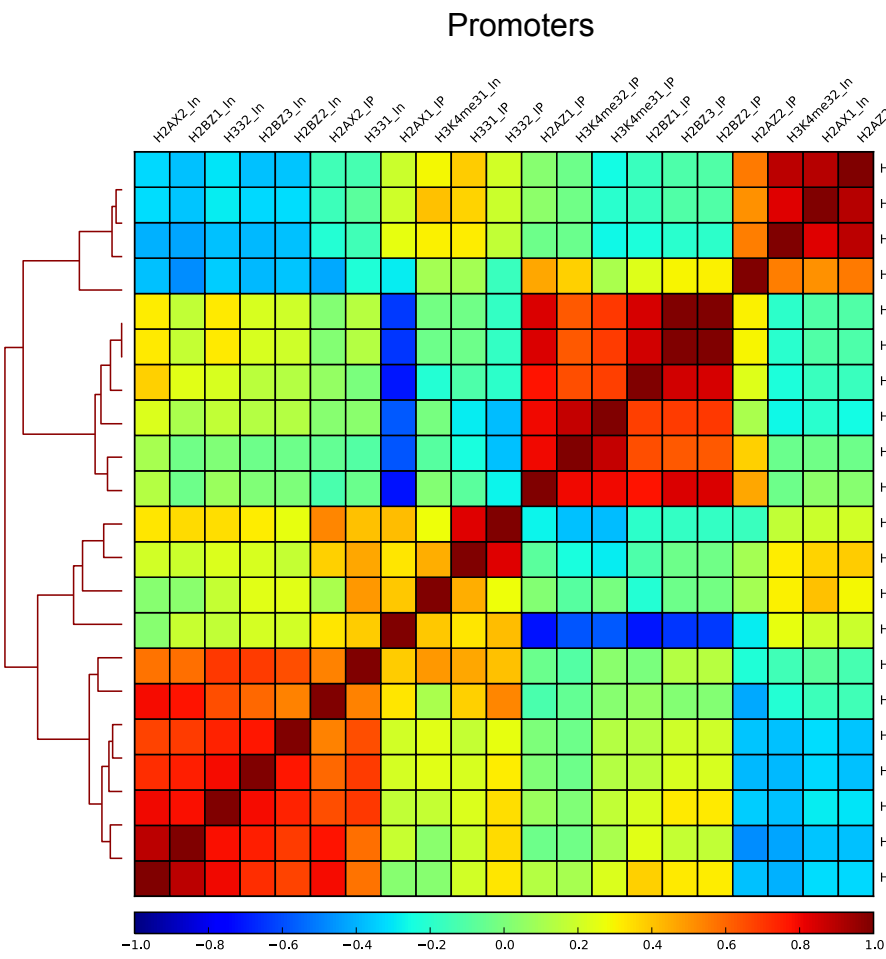

Supplement: Supplementary file 3 — Additional file 3. Correlation between ChIP-seq datasets. Clustered heat map of correlations between all ChIP-seq alignment files for: A. 1 kb bins genome-wide; B. Restricted to promoter regions only. Samples are indicated on the Axes (see additional file 1 for list) with IP indicating ChIP and In indicating input sample for each experimental sample. Color key indicating correlation coefficient (Spearman) indicated below (negative correlation in blue, positive correlation in red). [file 12864_2022_8338_MOESM3_ESM.pdf]

A

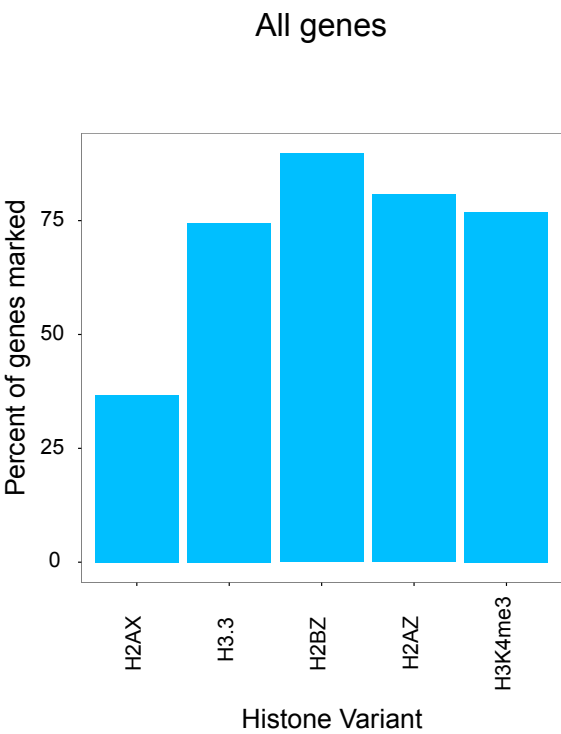

B

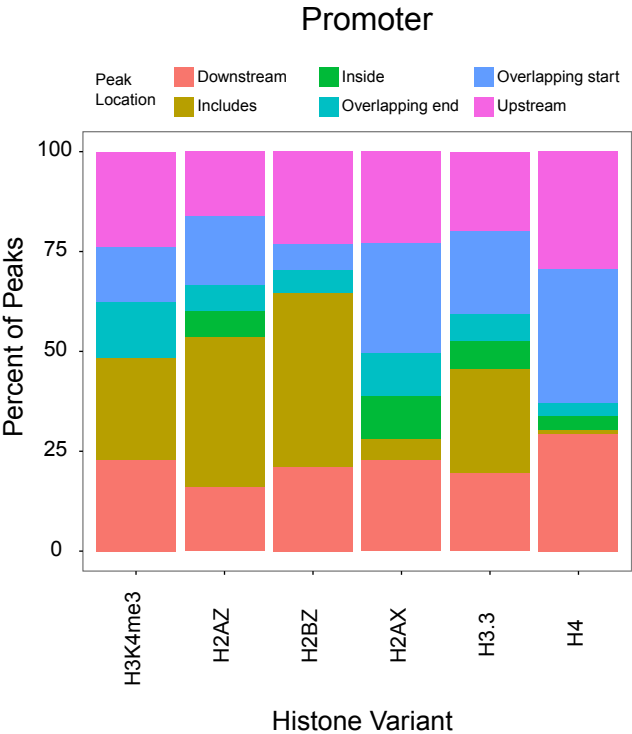

C

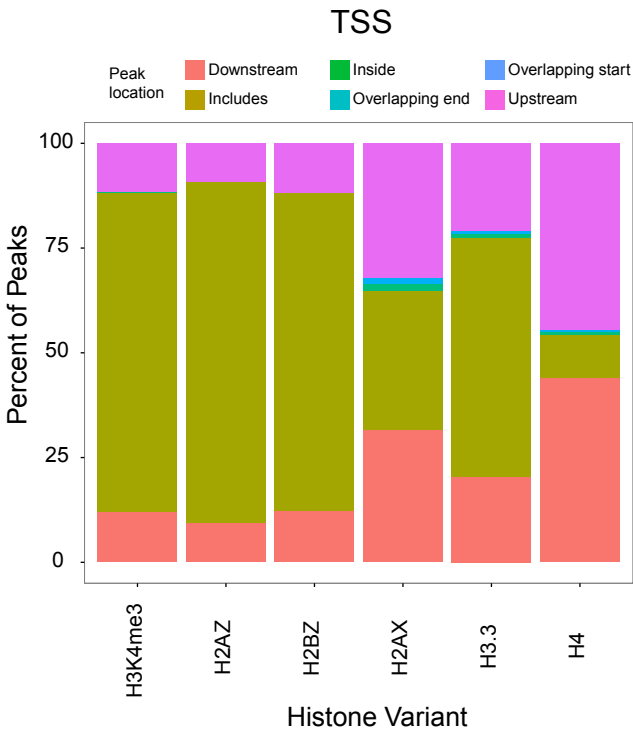

D

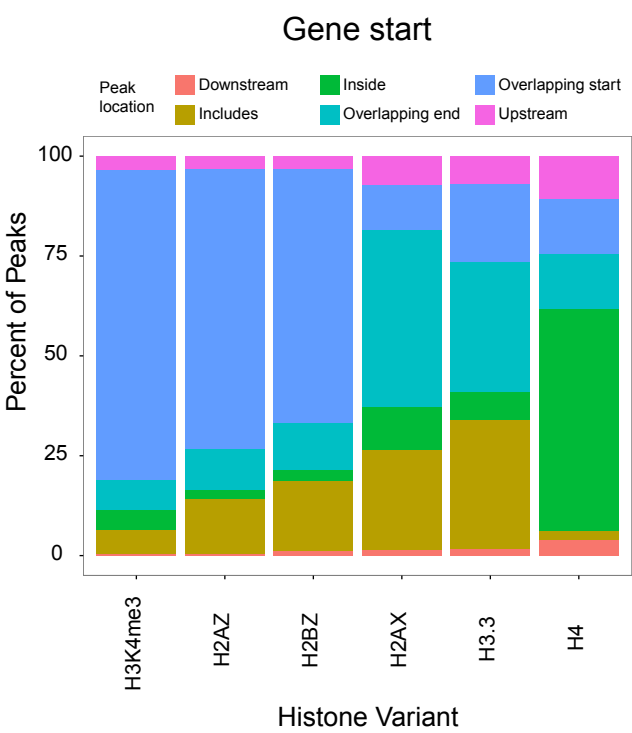

Supplement: Supplementary file 4 — Additional file 4. Distribution of histone variant ChIP-seq peaks over genes. A. Percentage of all annotated genes marked by histone variant peaks (regardless of location of peaks) is plotted. B, C, D. Plots depicting the percentage of ChIP-seq peaks with different locations of ChIP-seq peaks relative to the annotated promoter (1 kb upstream of transcriptional start site) (B), transcriptional start site (TSS) (C), gene start codon (D). [file 12864_2022_8338_MOESM4_ESM.pdf]

A

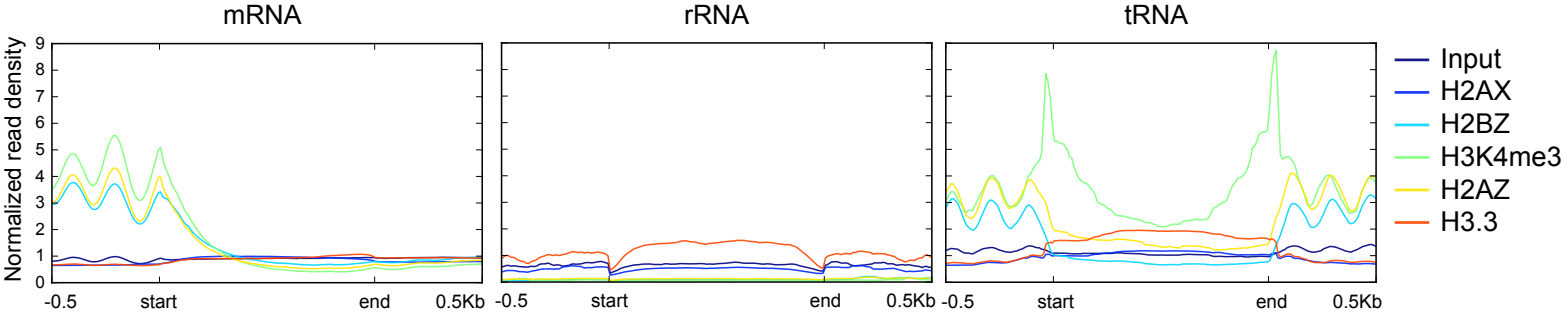

B

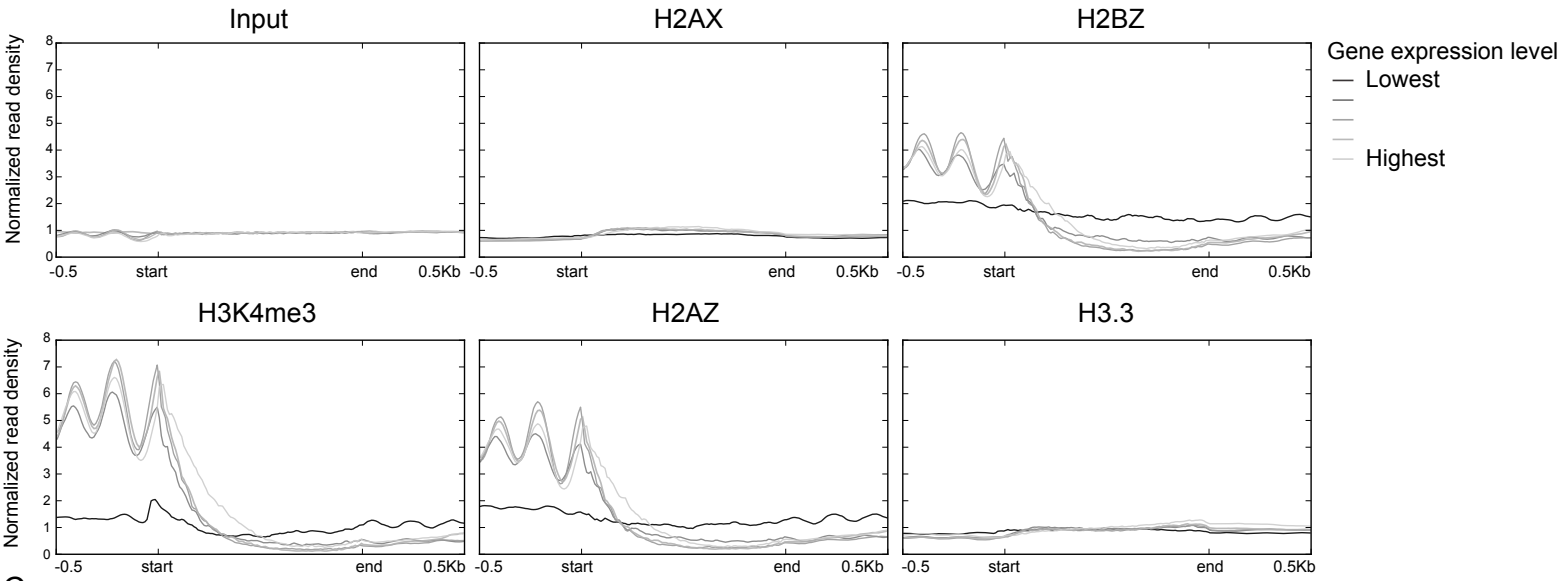

C

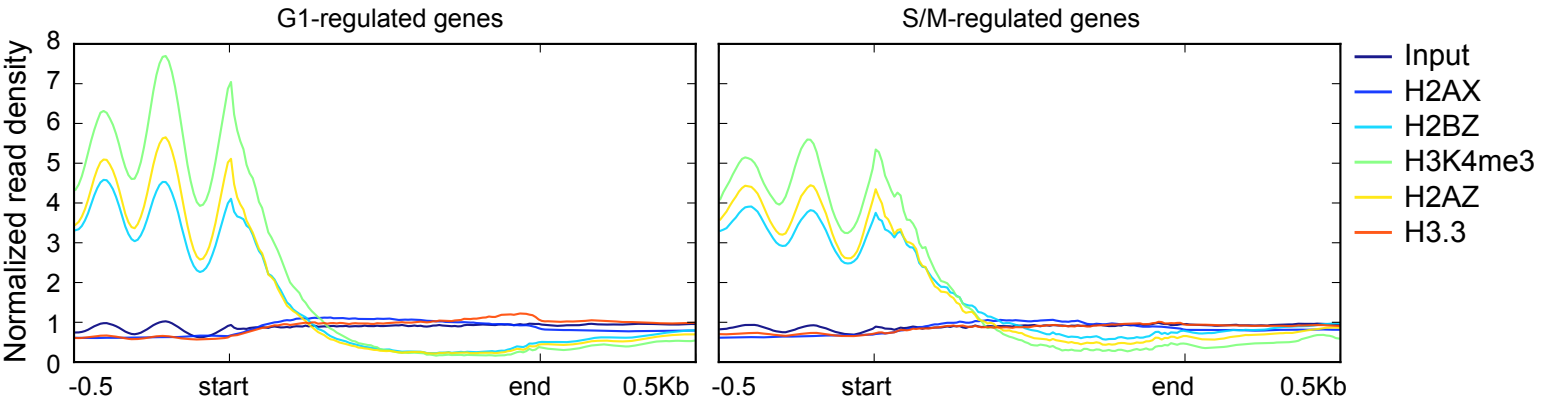

Supplement: Supplementary file 5 — Additional file 5. Average histone variant ChIP-seq profiles across different genomic features. Plots show normalized read density (experimental IP/input) for each ChIP-seq sample. A. RNA gene classes include: transcribed genes mRNA, ribosomal RNA rRNA, transfer RNA tRNA. B. Genes with different mRNA expression levels in tachyzoites. T. gondii genes were split into 5 equal groups based on their expression level in tachyzoites (determined from RNA-seq of T. gondii RH strain tachyzoites [31]). Average normalized ChIP-seq read densities were calculated over genes in each group and plotted. Lightest grey indicates highest quintile expression, whereas black indicates genes that had no detectable expression. C. G1- and S/M- regulated genes. T. gondii genes were previously classified those that are transcribed in accordance with G1 or S/M phase of cell cycle [29]. Average normalized ChIP-seq read densities were calculated for genes in each set and plotted. [file 12864_2022_8338_MOESM5_ESM.pdf]

Coverage 0-20% 40-60% 80-100% --- p = 0.05

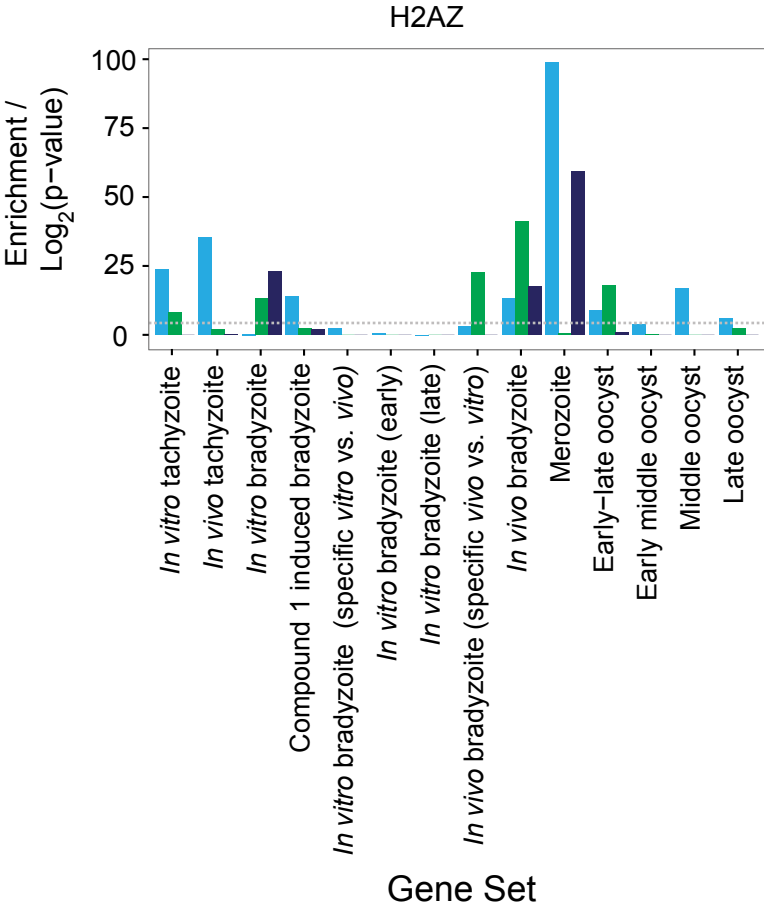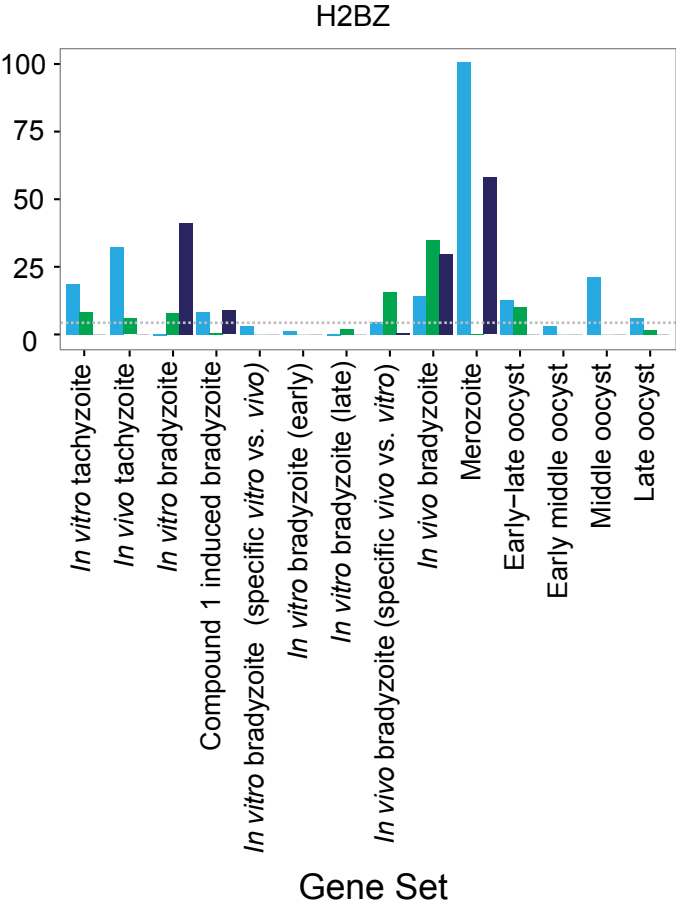

Supplement: Supplementary file 6 — Additional file 6. Stage-specific gene regulatory patterns of histone variants. Genes with different amounts of histone variant coverage were compared to sets of genes upregulated in different parasite life cycle stages (defined in [21, 29]. –log2(p-value) of enrichment is plotted for genes with different levels of coverage for H2AZ and H2BZ. p-value of < 0.05 in the hypergeometric test was considered significant and is indicated by the dotted grey line. [file 12864_2022_8338_MOESM6_ESM.pdf]
